# Development of panorama resolution phantom for comprehensive evaluation of the horizontal and vertical resolution of panoramic radiography

**DOI:** 10.1038/s41598-020-73639-3

**Published:** 2020-10-05

**Authors:** Han-Gyeol Yeom, Jo-Eun Kim, Kyung-Hoe Huh, Won-Jin Yi, Min-Suk Heo, Sam-Sun Lee, Soon-Chul Choi, Sang-Jeong Lee

**Affiliations:** 1 Department of Oral and Maxillofacial Radiology and Dental Research Institute, School of Dentistry, Seoul National University, 101 Daehak-ro, Jongno-gu, Seoul, 03080 Korea; 2 Department of Oral and Maxillofacial Radiology, School of Dentistry, Wonkwang University, Iksan, Korea; 3 Department of Biomedical Radiation Sciences, Seoul National University Graduate School of Convergence Science and Technology, Seoul, Korea

**Keywords:** Health care, Medical research

## Abstract

Panoramic radiography is the most commonly used equipment in the dental field, but there is no comprehensive standard about how to evaluate the spatial resolution of panoramic radiography. In this study, panorama resolution phantoms were developed for evaluation of horizontal and vertical resolution reflecting unique characteristics of panoramic radiography. Four horizontal resolution phantoms of staircase shape were designed to obtain images of horizontal lead line pairs in a 52 mm width. Four vertical resolution phantoms with vertical lead line pairs placed at an oblique angle were also designed. A phantom stand was made. Three machines were evaluated twice by two oromaxillofacial radiologists. The horizontal lead line pairs were readable over the entire measured area at the values of 1.88, 2.32, and 2.58 lp/mm for all machines. A readable area of horizontal 3.19 lp/mm was observed in the lingual side. In the vertical resolution phantoms, it was possible to read a narrower range. By using the panorama resolution phantoms and phantom stand, it was possible to evaluate the resolution of a wide buccolingual width in four significant areas. By evaluating the resolution of the vertical and horizontal compartments separately, it was possible to gain a better understanding of the obtained images.

## Introduction

Panoramic radiography is a technique that combines the principles of scanning and tomography^1,2^. In panoramic radiography, a horizontally narrow X-ray beam is used to scan the orofacial region, and the obtained X-ray image is recorded on a rotating single large film^1,3,4^. The radiographic image shows the body structures through the focal trough, image layer, or zone of sharpness of the machine^1,5,6^.

The focal trough is located in a vertical curved plane^1,3,7^. Image sharpness gradually decreases as the objects deviate from the center of the focal trough, and images are no longer identifiable on the radiograph^2,3^. This unique characteristic is of clinical importance as the radiologists can acquire only the images required for diagnosing the oral and maxillofacial region without overlapping structures^2^; however, the complexity of the process of image acquisition makes it difficult to evaluate the quality of panoramic radiography images using prevalent image quality evaluation methods.

The resolution of panoramic radiography has not been evaluated or controlled by reflecting the abovementioned unique characteristics of panoramic radiography. For evaluating the resolution of the majority of diagnostic radiographic machines, the images of lead line pairs are normally used^8^. However, owing to the unique characteristic in the process of obtaining images, various resolution values can be recorded according to the position of the resolution phantom even when using the same piece of panoramic radiography^1,2^.

In the “Acceptance tests-Imaging performance of dental X-ray equipment” issued by the International Electrotechnical Commission (IEC) in 2000, panoramic radiography was evaluated using a phantom including pairs of oblique lead lines measuring 1.6–3.0 lp/mm^9^. According to this standards, panoramic radiography resolution was evaluated by placing an oblique line pair phantom between the primary diaphragm and secondary diaphragm, but concrete mediolateral or anteroposterior locations were not specified. In 2009, the Deutsches Institut für Normung (DIN, German Institute for Standardization) proposed a minimal requirement value of 2.5 lp/mm to acquire images with acceptable diagnostic value in dental panoramic radiography but the criteria regarding which regions should be evaluated remain ambiguous^10^. Choi et al. proposed a method to evaluate the resolution values of panoramic radiography at various position by placing the horizontal lead line pairs on an arch shaped phantom stand^8^. However, this method did not sufficiently consider the changes of resolution values according to the changes of the buccolingual position.

What makes evaluating resolution of panoramic radiography more difficult is that the resolutions could be measured differently depending on which resolution phantoms were used, horizontal, vertical, or oblique type. In the previous studies about resolution of panoramic radiography, various types of lead line pairs(horizontal^8^, vertical^1–3^, and oblique line pairs^9,10^) were used without special criteria. It is easy to think that the oblique lead line pairs evaluates horizontal and vertical resolution together, but the horizontal and vertical elements of obtained image are affected completely differently by rotating image acquisition process, so they must be evaluated separately. Each results show different representative values of panoramic radiography and help to better understand the acquired images, but there has been no research focused on this distinction.

Panoramic radiography is the most commonly used equipment in the dental field, but there is no comprehensive standard about how to evaluate the spatial resolution of the equipment. Therefore, there is a need to establish a standard regarding how to measure and represent resolution of panoramic radiography by reflecting all these unique characteristics of the equipment.

The purpose of this study was to develop panorama resolution phantom for comprehensive evaluation of horizontal and vertical resolution of panoramic radiography. Vertical and horizontal lead line pairs were used separately, and various buccolingual positions in the incisor, premolar, molar, and temporomandibular joint regions of the panoramic radiography were evaluated. Finally, some results were represented in the form of resolution maps to make it easy to understand and apply the results clinically.

## Material and methods

### Development of two types of resolution phantoms

Horizontal resolution phantoms of staircase shape were designed to obtain images of horizontal lead line pairs in the region corresponding to a 52 mm width in 4-mm intervals. One phantom consisted of 13 steps, each containing a pair of horizontal lead line pairs of the same value. Four horizontal resolution phantom were made, each for the 1.88, 2.32, 2.58, and 3.19 lp/mm. These values were determined by IEC standard 4, which used lead line pairs of 1.6–3.0 lp/mm for the evaluation of panoramic radiography^9^. Nuclear Associates model 07-501 SER. NO.12913 (Fluke Co., Cleveland, OH, USA) was used. It was laser cut and placed at each step.

Four vertical resolution phantoms with vertical lead line pairs placed at an oblique angle to the same width of 52 mm were also designed, each for the 1.88, 2.32, 2.58, and 3.19 lp/mm. To mark the 4-mm interval, ligature wires were used at a boundary to mark it horizontally.

**Figure 1:**
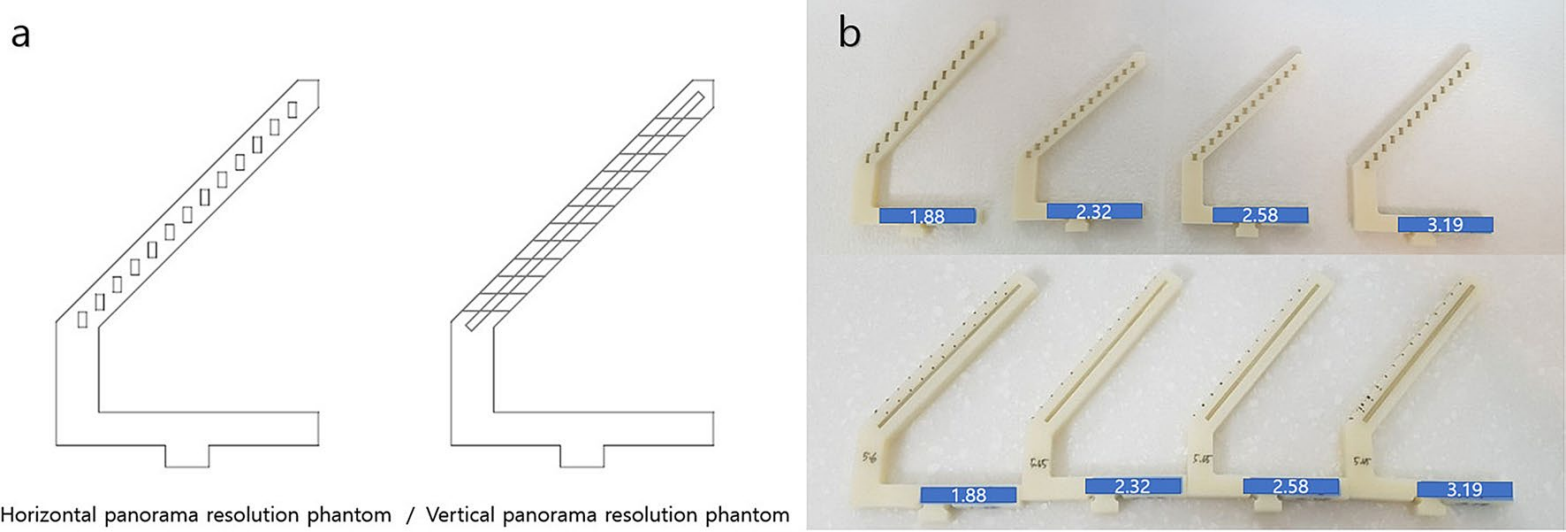
Final horizontal and vertical panorama resolution phantoms. (**a**) Final designs of panorama resolution phantoms. (**b**) Eight fabricated horizontal and vertical panorama resolution phantoms.

To overcome the limitation of overlapping images of the lead wire pairs at the lowest step due to the ghost image of the phantom stand placed on the opposite side or the actual image of the phantom itself, a space of 20 mm was left under the phantoms. The eight final resolution phantoms are shown in Fig. 1.

### Manufacturing of the phantom stand

To locate the prepared phantoms, an arch shaped phantom stand was constructed. It was designed to have square-shaped holes in four positions, incisor, premolar, molar, and temporomandibular joint (TMJ). The projective compartment in the bottom of panorama resolution phantom could be inserted into this square-shaped holes and stably positioned in the accurate location. The data of the incisor, premolar (central points of the cusps of mandibular first premolar and mandibular second premolar), molar (central points of the mesiobuccal cusp of mandibular first molar and the distobuccal cusp of mandibular second molar), and TMJ were determined by previously developed ball type panorama phantom^11^. To compensate for the position of the anteriorly inclined maxillary incisors, the hole was formed 5 mm posterior to the value obtained for the incisor.

**Figure 2:**
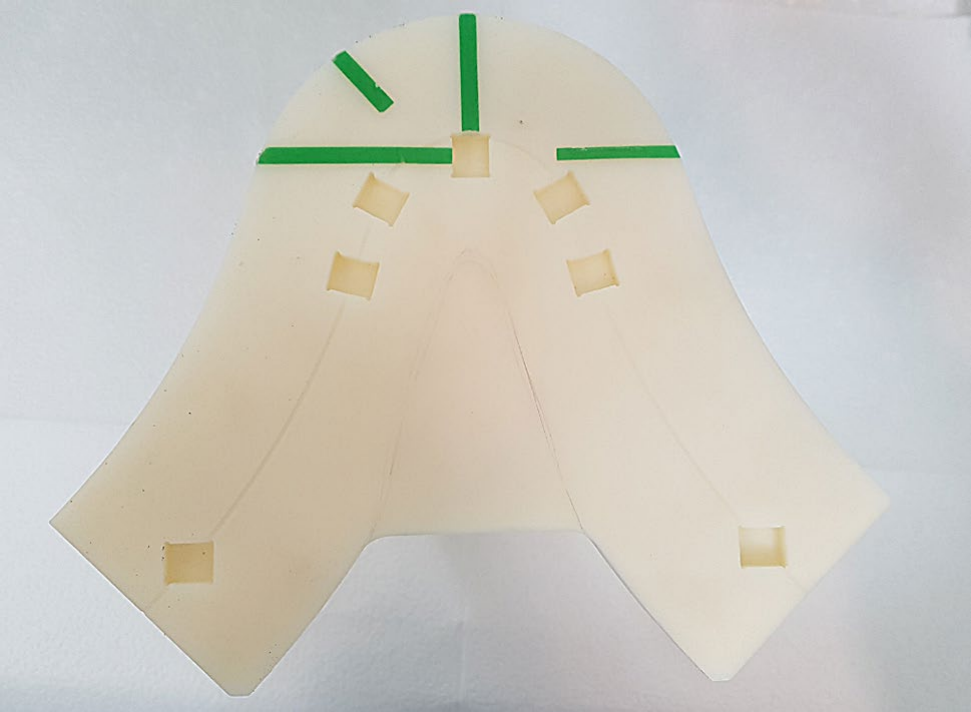
The fabricated phantom stand.

The angle of the hole for incisor was determined parallel to the midline of the phantom stand. The direction perpendicular to the line connecting the cusp of mandibular canine to the mesiobuccal cusp of mandibular first molar was used for the angle of the hole for premolar. The direction perpendicular to the line connecting the mesiobuccal cusp of mandibular first molar and the distobuccal cusp of 47 was used for the molar. The hole for mandibular condyle was made perpendicular to the line connecting the medial and lateral poles of the condyle, considering the horizontal angle of the condyle at 20°. The fabricated phantom stand is shown in Fig. 2.

### Acquisition of the resolution phantom images

The radiographic images of the phantoms were obtained using panoramic radiography OP-100 (Instrumentarium Dental, Tuusula, Finland), PCH-2500 (Vatech, Gyeonggi, Korea), and Rayscan α-P (Ray, Gyeonggi, Korea) at Seoul National University Dental Hospital, Seoul, South Korea. The images were obtained with the optimal parameters according to the user’s manual for imaging adult males, which were regularly used in the department. Parameters were 73 kVp, 10 mA, 17.6 s for OP-100. They were 73 kVp, 10 mA, 13.5 s for PCH-2500 and 73 kVp, 10 mA, 14.0 s for Rayscan α-P. The midpoint of the center line of the phantom stand was matched to the center of the incisive notch on panoramic radiography. A tripod water level was used to accurately position the phantom stand. The phantoms were positioned on the holes of the phantom stand.

The attenuation by the skull was reproduced with a 0.8-mm copper plate on the X-ray source, according to the recommendations of IEC standard 4^9^. To reproduce attenuation by soft tissue, a 6-mm aluminum plate should be attached to the front of the phantom^9^, but the entire phantom could not be covered uniformly due to structural limitations, so that it overlapped with the copper plate and placed in the X-ray source.

**Figure 3:**
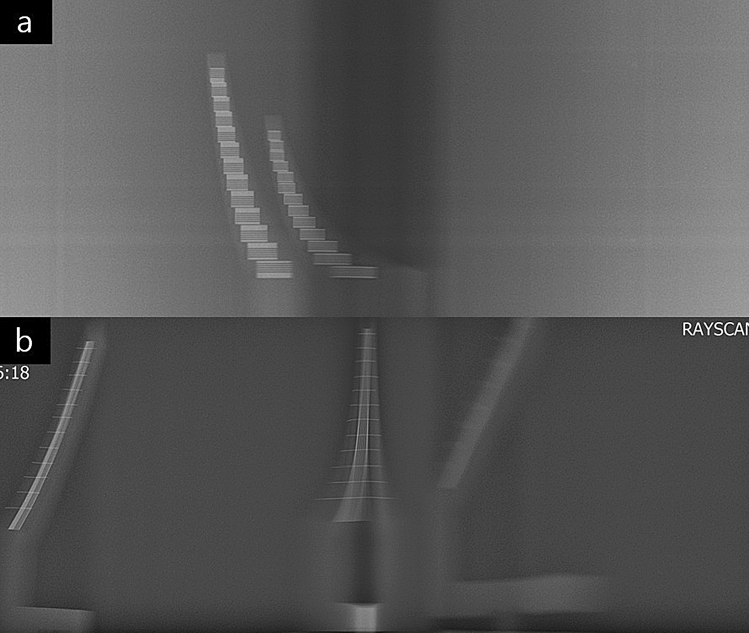
Examples of obtained phantom images. (**a**) Panoramic image of horizontal resolution phantoms by OP-100. (Premolar region: 2.32 lp/mm, Molar region: 1.88 lp/mm). (**b**) Panoramic image of vertical resolution phantoms by Rayscan α-P. (Incisor region: 2.58 lp/mm, TMJ region: 3.19 lp/mm).

In one acquisition, two different resolution phantoms were placed in two unaffected areas (incisor + TMJ or premolar + molar) (Fig. 3). To compensate for the error between the process of image acquisitions, images of the phantoms were obtained thrice in the same position. Finally, 24 images for horizontal resolution phantom and 24 images for vertical resolution phantom were obtained for each panoramic equipment.

### Evaluation of the images of the resolution phantoms

The images of resolution phantoms were evaluated twice by consensus of two oromaxillofacial radiologists. Following random arrangement of the images, the readable range numbers(1–13, numbered from lower area) were determined. When there was a discrepancy in the evaluation of the images, the reader who suggested ‘readable’ pointed at the readable part. Then, with the consent of another reader, the readability was determined. Disagreement did not occur in this way, so the disagreement rate was not recorded. To minimize error, images were rearranged randomly after 2 weeks and reevaluated. Intraclass correlation coefficient (ICC) was applied to evaluate the intra-observer reliability.

The images of one resolution phantom image were acquired thrice, and each image was read twice, resulting in six results for each resolution phantom at one region. The value that can be read more than four times was determined as the readable line pair value for the area.

## Results

The intra-observer analysis showed an excellent agreement between the two evaluation with a 2‐week interval. (ICC ≥ 0.90, p < 0.001) The horizontal resolution phantoms were readable at 52 mm, which is the entire evaluated area, at the values of 1.88, 2.32, and 2.58 lp/mm for all the three equipment. In the images obtained using the horizontal resolution phantom of 3.19 lp/mm, the readable width were narrower and observed in the lingual side of the arch. For OP-100, the readable width in the incisor, premolar, molar, and TMJ regions were 24, 12, 16 and 0 mm, respectively. For PCH-2500, the readable width were 8, 16, 0 and 8 mm, respectively. For Rayscan α-P, the readable width were 16, 0, 0 and 4 mm, respectively.

In the case of the vertical resolution phantoms, they were readable in a narrower range than the horizontal resolution phantoms, and the resulting area was observed along the center of the arch-shaped phantom stand.

The results for the readable range numbers of the horizontal and vertical resolution phantoms are shown in Table 1. The largest readable line pair value is indicated as the line pair value of the area.

**Table 1:** The results for the readable range numbers of the horizontal and vertical resolution phantoms.

Position	Incisor		Premolar		Molar		Temporomandibular joint	
Range number	Horizontal resolution (lp/mm)	Vertical resolution (lp/mm)	Horizontal resolution (lp/mm)	Vertical resolution (lp/mm)	Horizontal resolution (lp/mm)	Vertical resolution (lp/mm)	Horizontal resolution (lp/mm)	Vertical resolution (lp/mm)
**a. OP-100**								
1	3.19	×	3.19	×	3.19	×	2.58	2.58
2	3.19	×	3.19	×	3.19	×	2.58	2.58
3	3.19	×	3.19	×	3.19	1.88	2.58	2.58
4	3.19	2.58	2.58	2.32	3.19	2.32	2.58	2.32
5	3.19	3.19	2.58	2.58	2.58	2.58	2.58	1.88
6	3.19	2.58	2.58	2.58	2.58	2.58	2.58	1.88
7	2.58	×	2.58	1.88	2.58	2.58	2.58	1.88
8	2.58	×	2.58	×	2.58	2.32	2.58	1.88
9	2.58	×	2.58	×	2.58	×	2.58	×
10	2.58	×	2.58	×	2.58	×	2.58	×
11	2.58	×	2.58	×	2.58	×	2.58	×
12	2.58	×	2.58	×	2.58	×	2.58	×
13	2.58	×	2.58	×	2.58	×	2.58	×
**b. PCH-2500**								
1	2.58	×	2.58	1.88	2.58	3.19	3.19	2.58
2	2.58	2.32	2.58	3.19	2.58	3.19	3.19	2.58
3	3.19	3.19	3.19	3.19	2.58	3.19	2.58	2.58
4	3.19	3.19	3.19	3.19	2.58	3.19	2.58	2.32
5	2.58	2.58	3.19	2.58	2.58	2.58	2.58	2.32
6	2.58	2.58	3.19	2.58	2.58	2.58	2.58	1.88
7	2.58	2.58	2.58	2.58	2.58	2.58	2.58	1.88
8	2.58	2.58	2.58	2.58	2.58	2.32	2.58	1.88
9	2.58	2.58	2.58	2.58	2.58	2.32	2.58	×
10	2.58	1.88	2.58	1.88	2.58	2.32	2.58	×
11	2.58	×	2.58	1.88	2.58	2.32	2.58	×
12	2.58	×	2.58	1.88	2.58	2.32	2.58	×
13	2.58	×	2.58	1.88	2.58	2.32	2.58	×
**c. Rayscan α-P**								
1	3.19	×	2.58	×	2.58	×	3.19	2.32
2	3.19	×	2.58	×	2.58	×	2.58	2.32
3	3.19	×	2.58	×	2.58	1.88	2.58	2.32
4	3.19	2.58	2.58	2.32	2.58	3.19	2.58	2.58
5	2.58	3.19	2.58	2.58	2.58	3.19	2.58	×
6	2.58	2.58	2.58	2.58	2.58	2.58	2.58	×
7	2.58	1.88	2.58	1.88	2.58	1.88	2.58	×
8	2.58	1.88	2.58	×	2.58	×	2.58	×
9	2.58	×	2.58	×	2.58	×	2.58	×
10	2.58	×	2.58	×	2.58	×	2.58	×
11	2.58	×	2.58	×	2.58	×	2.58	×
12	2.58	×	2.58	×	2.58	×	2.58	×
13	2.58	×	2.58	×	2.58	×	2.58	×

×: No line pair value could be read.

**Figure 4:**
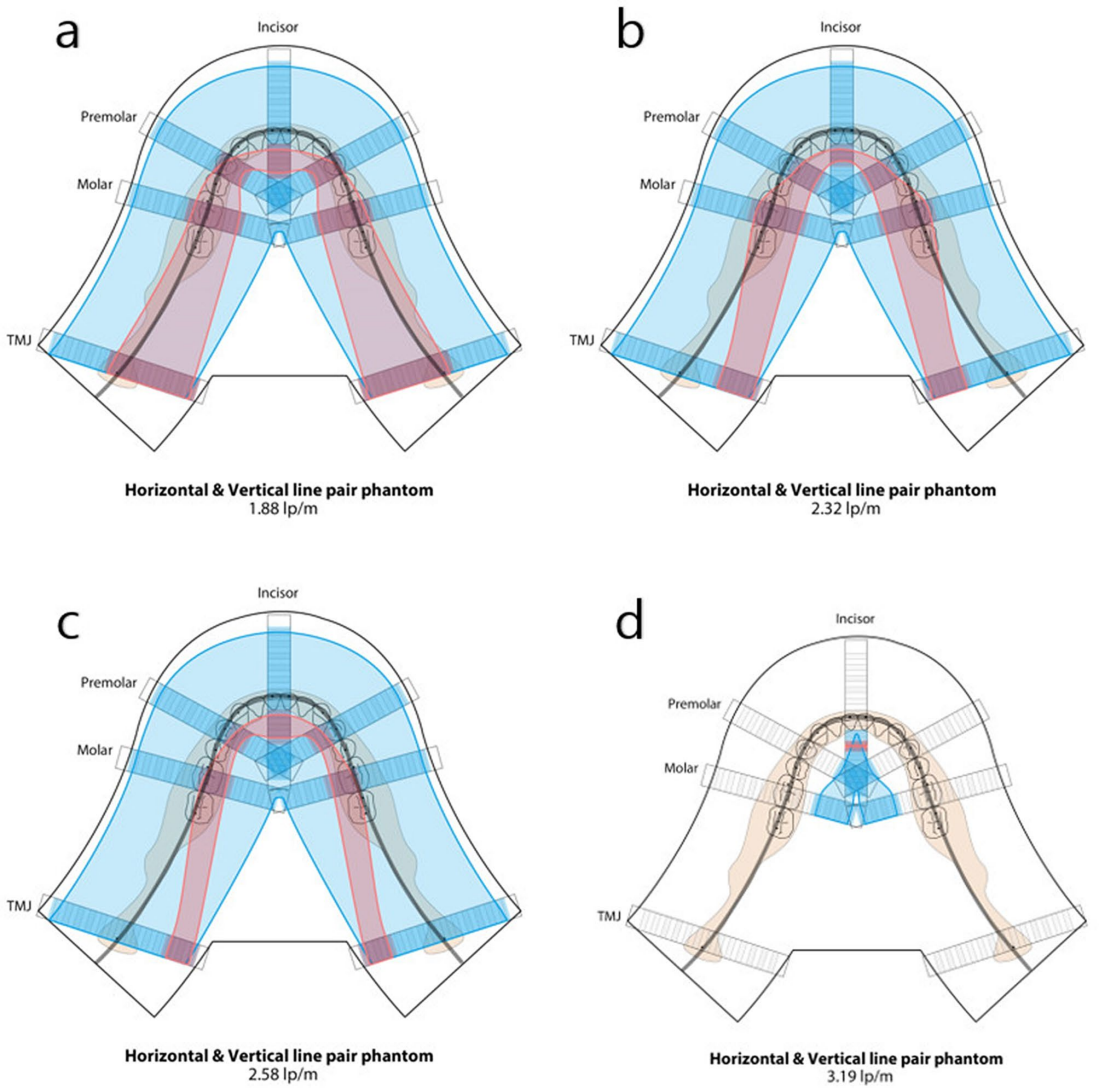
Resolution illustration for OP-100. The final results of the readable areas evaluated using the horizontal and vertical resolution phantoms (Blue area: evaluated using horizontal resolution phantom, Red area: evaluated using vertical resolution phantom). (**a**) 1.88 lp/mm, (**b**) 2.32 lp/mm, (**c**) 2.58 lp/mm, (**d**) 3.19 lp/mm.

As an example to help clinicians understand, the final results of the readable areas of OP-100 are drawn on the arch-shaped illustration in Fig. 4.

## Discussion

Obtaining accurate spatial resolution of panoramic radiography equipment is important not only for managing the quality of the equipment, but also for the clinician's ability to understand and accurately read and diagnose images.

In this study, the resolution value using horizontal or vertical resolution phantoms resulted in significant differences owing to the unique characteristics of panoramic radiography in the process of obtaining radiographic images. In addition, even when using the same type of lead line pair, different resolution values were measured depending on the anteroposterior or mediolateral position. These results show that one of the vertical or horizontal line pair phantoms as well as one specific position is not sufficient for use as a representative value for the resolution of the panoramic radiography.

To evaluate resolution of panoramic radiography accurately, we suggest that two points should be considered and specified.

First, the location where the lead line pairs will be positioned should be specified.

According to the IEC regulations, evaluating the resolution of panoramic radiography is achieved by placing an oblique line pair phantom between the primary and secondary diaphragm^9^. But mediolateral or anteroposterior location of the phantom was not specified. Based on this IEC regulation, the resolution of the machine could be measured at any portion having the highest resolution rather than measured at the actual jaw position. It could be a clinically insignificant representative value. As the values of the readable lead line pairs were measured differently for each region and for each buccolingual side of the region, the results need to be evaluated and studied independently. And the further research to set recommendation of resolution values should be conducted for each positions, rather than for one representative position.

Second, each horizontal and vertical resolution phantom should be used at same position and each results should be presented separately.

As the film and the X-ray source move horizontally, the information in the horizontal direction is relatively more deformed, whereas the information in the vertical direction is relatively free from influence^1^. In this study, the difference in resolution values evaluated using two types of resolution phantom well represented this characteristic of the panoramic radiography. Evaluating the resolution using the vertical resolution phantom is using the horizontal difference (horizontal information) between the vertically arranged lead lines, so it shows more sensitive result according to the changes of the buccolingual position. By contrast, evaluating the resolution using horizontal resolution phantom, which determines the resolution by the vertical difference between horizontally arranged lines, results in a relatively less sensitive value. This result is encouraging and will assist in understanding the acquired image and evaluating the quality of the image.

Panoramic radiography is not free from the influence of overlapping of various peripheral structures, and information regarding the resolution of the horizontal information and vertical information of the object at each part is also important for exact diagnosis. This provides additional information to assist in understanding and diagnosing lesions that may be present at various locations.

For example, in the center region of the premolar, the highest readable horizontal resolution phantom was 2.58 lp/mm and that of the vertical resolution phantom was 1.88 lp/mm for OP-100. Therefore, for a lesion located at the premolar position, the horizontal compartment is read with the resolution of 1.88 lp/mm and the vertical compartment is read with resolution of 2.58 lp/mm. This means that, even if the lesion has same clear margin in three dimensions, it can be visualized more clearly vertically. This fact is already known to an extent, but using this method has made it clear that it can be quantified according to the location.

The majority of dental clinics use panoramic radiography; however, owing to the complexity of the acquisition of panoramic radiography, it is difficult to understand and apply the evaluated quality of this equipment to clinical diagnosis. Nevertheless, accurate management of the quality of panoramic radiography and accurate identification of the information obtained, such as the horizontal and vertical resolutions in each position of the patient, will assist in providing an accurate diagnosis using imaging. The resolution illustration example from our study will help to intuitively understand the resolution of the panoramic image.

## Conclusion

In panoramic radiography, the resolution evaluated using horizontal and vertical resolution phantoms resulted in significant differences owing to the unique characteristics of panoramic radiography in the process of obtaining radiographic images.

By using the proposed horizontal and vertical resolution phantoms and phantom stand, it was possible to evaluate the resolution of a wide buccolingual width of 52 mm in four significant parts of the oral and maxillofacial region. And it is possible to gain a better understanding of the obtained radiographic images by evaluating the resolution of the vertical and horizontal compartments separately.

Because the resolution of several areas were evaluated specifically, it is possible to show resolution maps of panoramic radiography for comprehensive understanding of general dentists who own the evaluated panoramic machine.
